# H3K9me2 regulation of BDNF expression via G9a partakes in the progression of heart failure

**DOI:** 10.1186/s12872-022-02621-w

**Published:** 2022-04-19

**Authors:** Fang Yan, Ziying Chen, Wei Cui

**Affiliations:** 1 Department of Cardiac Surgery, Hebei Medical University, Shijiazhuang, 050011 Hebei People’s Republic of China; 2 Department of Cardiac Surgery, The Second Hospital of Hebei Medical University, No. 215, Heping West Road, Xinhua District, Shijiazhuang, 050000 Hebei People’s Republic of China; 3 Department of Cardiology, The Second Hospital of Hebei Medical University, No. 215, Heping West Road, Xinhua District, Shijiazhuang, 050000 Hebei People’s Republic of China

**Keywords:** G9a, BDNF, Heart failure, TrkB signaling pathway, H3K9me2

## Abstract

**Background:**

Heart disease is a major cause of mortality in developed countries. The associated pathology is mainly characterized by the loss of cardiomyocytes that contributes to heart failure (HF). This study aims to investigate the mechanism of euchromatic histone lysine methyltransferase 2 (EHMT2, also term G9a) in HF in rats.

**Methods:**

Differentially expressed mRNAs in HF were screened using GEO database. Sera from subjects with or without HF were collected, and PCR was performed to detect the G9a expression. G9a was downregulated in cardiomyocytes exposed to oxygen–glucose deprivation (OGD), followed by CCK8, flow cytometry, colorimetric method, and western blot assays. Established HF rats were delivered with lentiviral vectors carrying sh-G9a, and TTC staining, HE staining, TUNEL, ELISA, and western blot were performed. The regulation of G9a on the downstream target BDNF was investigated by RT-qPCR, Western blot, and ChIP-qPCR. Finally, rescue experiments were carried out to substantiate the effect of G9a on cardiomyocyte apoptosis and injury via the BDNF/TrkB axis.

**Results:**

G9a was overexpressed, whereas BDNF was downregulated in HF. Knockdown of G9a inhibited apoptosis and injury in OGD-treated cardiomyocytes and attenuated the extent of HF and myocardial injury in rats. Silencing of G9a promoted BDNF transcription by repressing H3K9me2 modification of the BDNF promoter. Further depletion of BDNF partially reversed the effect of sh-G9a in alleviating cardiomyocyte apoptosis and injury by inhibiting the TrkB signaling pathway.

**Conclusion:**

G9a inhibits BDNF expression through H3K9me2 modification, thereby impairing the TrkB signaling pathway and exacerbating the development of HF.

**Supplementary Information:**

The online version contains supplementary material available at 10.1186/s12872-022-02621-w.

## Background

Heart failure (HF) is a syndrome affecting more than 26 million people worldwide and the number is increasing [1]. HF is a shared chronic phase of cardiac functional damage secondary to many aetiologies, and patients with HF experience many symptoms that distress their quality of life, including fatigue, poor exercise tolerance, and fluid retention [2]. The mammalian heart has a very restricted capacity to regenerate after injury, and the lost cells are replaced by a fibrotic scar, which is followed by remodeling of the surrounding myocardium, including thickening (hypertrophy) and stiffening (fibrosis) of the left ventricular (LV) wall, and finally contributes to impaired cardiac function [3]. It is, therefore, of great importance to inhibit myocardial fibrosis to improve the treatment and outcomes of HF.

Epigenetics are mechanisms modulating gene expression independently of alterations to DNA sequences, which can be grouped into four main categories: DNA methylation, histone modifications, chromatin remodeling, and noncoding RNA regulations [4]. Back to 2009, trimethylation of histone H3 on lysine-9 (H3K9me3) was found to markedly affect cardiomyocytes in association with the development of HF in a rat disease model [5]. In the present study, we observed that euchromatic histone lysine methyltransferase 2 (EHMT2, also term G9a) was significantly overexpressed in the human endomyocardial biopsies of patients with dilated cardiomyopathy relative to 7 individuals with normal LV ejection fraction (LVEF) in the GEO database, indicating its possible in HF. H3K9me is a highly conserved histone post-translational modification, and G9a-mediated H3K9me2 is highly linked with transcriptional silencing [6]. Interestingly, G9a has been established to promote cardiac hypertrophy by inhibiting antihypertrophic genes [7]. However, many questions still remain untouched and unanswered regarding the role of G9a in HF. Early life stress has recently been indicated to contribute to increased H3K9me2 expression and decreased brain-derived neurotrophic factor (BDNF) expression in the hippocampus of rats, underlining the possible association between BDNF expression and H3K9me2 modification in its promoter [8]. BDNF has extensive roles by binding to tropomyosin-related kinase receptor B (TrkB), and the BDNF/TrkB pathway is found closely related to the outcome of cardiovascular diseases, including HF [9]. Patients with coronary artery disease exhibited significantly lower plasma BDNF levels than those of control patients [10]. Moreover, BDNF enhanced the skeletal muscle anti-atrophic effect of exercise training in mice with HF [11]. Therefore, our present study focused on the role of G9a and the associations between G9a and BDNF in the rats with HF and rat cardiomyocytes H9C2 with oxygen–glucose deprivation (OGD).

## Methods

### Blood collection

Blood samples of 40 patients with confirmed HF (collected within 6 h of the onset) and that of 40 healthy subjects were collected from January 2019 to October 2020 at the Second Hospital of Hebei Medical University. The differences in gender composition and age between the two groups were not significant. The main exclusion criteria were patients with renal failure (serum creatinine > 176 μmol/L). Within 30 min after blood collection, peripheral blood from patients with HF and healthy subjects was collected in 10 mL ethylenediaminetetraacetic acid anticoagulation tubes and centrifuged at 3000 g and 4 °C for 10 min. Plasma was separated immediately after centrifugation to obtain the serum which was stored at -80 °C for subsequent analysis. The study was performed with the approval of the institutional Ethics Committee of the Second Hospital of Hebei Medical University (approval number: 2019-R005). The studies abide by the *Declaration of Helsinki* principles. The relevant rules and regulations of the institutional Ethics Committee of the Second Hospital of Hebei Medical University were strictly followed, and written informed consent was obtained from all participants.

### Cell culture

Rat cardiomyocytes H9C2 (CRL-1446) were from American Type Culture Collection (Manassas, VA, USA). H9C2 cells were cultured in DMEM containing 10% FBS, 100 U/mL penicillin, and 100 µg/mL streptomycin (Thermo Fisher Scientific Inc., Waltham, MA, USA) at 37 °C and 5% CO_2_.

Short hairpin RNA (sh)-G9a, sh-BDNF and negative control (sh-NC) were designed by Shanghai GenePharma Co., Ltd. (Shanghai, China). The constructs sh-G9a, sh-BDNF and sh-NC were transfected or co-transfected into well-grown H9C2 cells using the Lipofectamine 2000 transfection kit (Thermo Fisher Scientific) according to the instructions. At 12 h after transfection, the medium was replaced with fresh medium, and the H9C2 cells were incubated for another 24 h for subsequent experiments. For pathway blockage, the H9C2 cells were treated with a TrkB inhibitor K252a (100 ng/mL, Life Technologies, Carlsbad, CA, USA) or the same dose of dimethylsulfoxide (DMSO) for 30 min after sh-G9a transfection. When H9C2 cell confluence reached 80% again, the H9C2 cells were washed with phosphate-buffered saline. For OGD treatment, the H9C2 cells were incubated in serum-free medium in an anaerobic chamber containing 95% N_2_ and 5% CO_2_ for 6 h at 37 °C [12], followed by growth in DMEM containing 10% FBS at 37 °C and 5% CO_2_ for 12 h. The H9C2 cells in the control group were not treated anaerobically.

### Animals

All animal experiments were approved by the Experimental Animal Ethic Committee of the Second Hospital of Hebei Medical University (approval number: 2020–1-015) and conformed to the ARRIVE guidelines and the Guide for the Care and Use of Laboratory animals published by the US National Institutes of Health. Forty-eight healthy male Sprague–Dawley rats (205 ± 16 g, Vital River, Beijing, China) were acclimatized for 7 days in a room (temperature 23 ± 2 °C; humidity 50–70%) with a 12:12-h light–dark cycle and allowed free access to standard laboratory chow and tap water.

Animals were randomly grouped by random numbers: the animals were assigned a random number and grouped according to the random number. G*Power software (https://stats.oarc.ucla.edu/other/gpower/) was used to obtain the minimum sample size to ensure statistical significance between groups. Using a one-way ANOVA with α = 0.05 and power = 0.8, we found that the minimum number of animals in each group was 4. Therefore, 6 rats were used per group for myocardial histological and cytokine expression analyses. HF in rats is induced by ligation of the left anterior descending (LAD) between the left auricle and the pulmonary artery. In brief, rats were anesthetized by intraperitoneal injection with a mixture of xylazine (5 mg/kg) and ketamine (100 mg/kg). The surgery was performed under aseptic conditions. After sterilization of the chest cavity, thoracotomy was performed via the fourth intercostal space, and the pericardium was opened. The left coronary artery was quickly ligated with a single nylon suture. The chest cavity was closed and gently massaged to restore the voluntary breathing. Finally, the incision was closed layer by layer. Sham-operated animals underwent a similar procedure except for ligation of the LAD. Postoperatively, 400,000 U of penicillin was administered to all rats to prevent infection.

Rats were randomly divided into 8 groups (n = 6): the sham, HF, sh-NC, sh-G9a, sh-G9a + sh-NC, sh-G9a + sh-BDNF, sh-G9a + DMSO, sh-G9a + K252a groups. sh-G9a and sh-BDNF were subcloned into the lentiviral plasmid LV-GFP, and approximately 1 × 10^9^ TU/mL lentiviruses (100 μL/rat) were injected into the left ventricle at five sites around the infarct border after LAD ligation [13]. K252a was dissolved in 25% DMSO (final concentration 10 µg/mL) and injected at a dose of 100 µg/kg/day for 7 consecutive days prior to LAD ligation (with DMSO as control) [14].

After 4 weeks, the cardiac function of rats was assessed by echocardiography. The rats were euthanized (intraperitoneal injection of sodium pentobarbital at 150 mg/kg), and serum and cardiac tissues were obtained for subsequent experiments.

### RT-qPCR

Total RNA was isolated from sera from HF patients and healthy subjects, cells, and rat heart tissues using TRIzol (Invitrogen, Carlsbad, CA, USA). Trizol was mixed with serum in equal volume and left to stand for 10 min, and then chloroform was added. After a 4000-g centrifugation for 15 min, the upper phase was centrifuged again with an equal volume of isopropanol. After the addition of 70% ethanol, the samples were centrifuged again. The precipitate was dried for 5 min and then dissolved by adding DEPC water to obtain RNA. RNA concentration was measured using a Nanodrop spectrophotometer (ND-100, Thermo Fisher Scientific). Total RNA was reverse transcribed to cDNA using a reverse transcription kit (Thermo Fisher Scientific). Quantitative PCR was performed using the KAPA SYBR FAST qPCR kit (Kapa Biosystems, Wilmington, MA, USA) and specific primers (Takara Biotechnology Ltd., Dalian, Liaoning, China) in a 7900HT fast real-time PCR system (Applied Biosystems, Inc., Foster City, CA, USA). The specific sequences of the primers are shown in Table 1. The expression of gene transcripts was quantified with the 2^−ΔΔCt^ relative quantification method that was normalized to glyceraldehyde-3-phosphate dehydrogenase (GAPDH).

**Table 1:** Forward and reverse primers for RT-qPCR

Gene	Sequences (5′-3′)
hsa-G9a-F	GGTGAACAACCACCTGGAGGTA
hsa-G9a-R	AGGCTGACCATCTCCAAGTTCC
rno-G9a-F	GGAGCCAACATCAATGCCGTAG
rno-G9a-R	TAGACAGGTGGAGCCATCCTCT
hsa-BDNF-F	CATCCGAGGACAAGGTGGCTTG
hsa-BDNF-R	GCCGAACTTTCTGGTCCTCATC
rno-BDNF-F	GGCTGACACTTTTGAGCACGTC
rno-BDNF-R	CTCCAAAGGCACTTGACTGCTG
hsa-GAPDH-F	GTCTCCTCTGACTTCAACAGCG
hsa-GAPDH-R	ACCACCCTGTTGCTGTAGCCAA
rno-GAPDH-F	CATCACTGCCACCCAGAAGACTG
rno-GAPDH-R	ATGCCAGTGAGCTTCCCGTTCAG

hsa, homo sapiens; rno, rattus norvegicus; G9a, euchromatic histone lysine methyltransferase; BDNF, brain-derived neurotrophic factor; GAPDH, glyceraldehyde-3-phosphate dehydrogenase; F, forward; R, reverse

### Western blot

Myocardial tissues and cells were collected and lysed using ultrasonic cell disruption on ice in radioimmunoprecipitation assay lysis buffer (Beyotime, Shanghai, China) for 30 min. The samples were centrifuged at 12,000 × g for 30 min at 4 °C to collect the supernatant. Protein concentrations were determined using a BCA protein assay kit (Sigma-Aldrich Chemical Company, St Louis, MO, USA). Protein lysates (30 μg) were loaded onto 12% sodium dodecyl sulfate–polyacrylamide gels for electrophoresis and transferred to polyvinylidene fluoride membranes (Millipore, Bedford, MA, USA). One hour of blocking was done at room temperature with 5% BSA (Gibco, Carlsbad, CA, USA). Membranes were incubated at 4 °C overnight with primary antibodies as follows: G9a (1:1000, ab185050, Abcam, Cambridge, UK), BDNF (1:2000, GTX132621, GeneTex, Inc., Alton Pkwy Irvine, CA, USA), H3K9me2 (1:1000, GTX54102, GeneTex), α-SMA (1:2000, GTX100034, GeneTex), FN1 (1:1000, ab268020, Abcam), p-TrkB (1:1000, ab229908, Abcam), TrkB (1:1000, GTX54857, GeneTex), GAPDH (1:10,000, ab181602, Abcam). After a wash, blots were incubated with goat anti-rabbit IgG-horseradish peroxidase (1:5000, ab6721, Abcam) for 60 min at room temperature. The blot was visualized with an ECL kit (Beyotime) and quantified with Quantity One software (Bio-Rad Laboratories, Hercules, CA, USA).

### Cell counting kit (CCK-8)

The H9C2 cells at the logarithmic growth phase were plated in a 96-well plate with 5,000 cells/well, and 100 μL of 0.25% trypsin was supplemented to each well to dissociate and resuspend the cells. The plates were incubated at 37 °C and 5% CO_2_ for 72 h, and 10 μL CCK8 solution (Beyotime) was supplemented to each well. The plates were incubated for 60 min at 37 °C in an incubator, and the optical density (OD) value at 450 nm was measured using a microplate reader (ELx800, Omega Bio-tek Inc, Norcross, GA, USA).

### Flow cytometry

We measured the apoptosis rate of H9C2 cells by flow cytometry using Annexin V-fluorescein isothiocyanate (FITC) Apoptosis Kit (Beyotime) as per the manufacturer's protocol. Briefly, cells at logarithmic growth phase were harvested, and the cell concentration was adjusted to 2 × 10^6^ cells/mL in phosphate-buffered saline. The cell suspension (200 μL) was centrifuged at 300 g for 10 min at 4 °C, and the supernatant was discarded. The cells were resuspended in 200 µL labeling buffer and incubated with 10 µL Annexin V-FITC and 5 µL propidium iodide (PI) for 15 min at room temperature in the darkness. Flow cytometry analysis was performed by FACScan flow cytometry (BD Biosciences, San Jose, CA, USA), and FlowJo software (Tree Star, Ashland, OR, USA) was applied to quantify the percentage of apoptotic cells.

### Lactate dehydrogenase (LDH) assay

After being seeded in the 96-well plate at 2 × 10^4^ cells/well, a total of 10 μL cell culture medium was aspirated at 48 h after transfection. The samples were diluted at 1:10 with LDH storage buffer containing 200 μM Tris–HCl (pH = 7.3), 10% glycerol and 1% BSA and analyzed using the LDH-Glo cytotoxicity kit (Promega Corporation, Madison, WI, USA).

### Echocardiographic measurements

Four weeks after the HF model establishment, the rats were anesthetized with a mixture of xylazine (5 mg/kg) and ketamine (100 mg/kg) and immobilized in a supine position on a wooden board. Echocardiography was conducted using a small animal ultrasound imaging system (VisualSonics, Toronto, ON, Canada) to assess LV end-diastolic volume (LVEDV) and LV end-systolic volume (LVESV), left ventricular end-diastolic internal diameter (Dd) and left ventricular end-systolic internal diameter (Ds). In order to standardize the experimental technique for echocardiographic measurements, the ventricular segments were first divided and limited, and the sampling site was ensured to be in the middle of each segment of the myocardium. Meanwhile, the size of the sampling area was standardized. The data were analyzed and averaged after superimposing at least three consecutive cardiac cycles using the composite imaging technique. LVEF and LV fractional shortening (LVFS) were then calculated, where LVEF = (LVEDV-LVESV) × 100% and LVFS = (Dd-Ds)/Dd × 100%.

### Triphenyltetrazolium chloride (TTC) staining

Evans-Blue staining (2.0% solution in 1 mL) was injected into the coronary circulation through a carotid catheter. After euthanasia, the hearts of rats were rapidly excised, stored at -80 °C, cut into 1-mm thick sections, and counter-stained at 37 °C for 15 min with 1% (wt/vol) TTC solution (Sigma). Representative images were captured. Image-Pro Plus software was used to assess the volumes of non-ischemic (area not at risk, ANAR), ischemic (area at risk, AAR), and infarcted (INF) regions, and the results were considered as INF/AAR × 100%.

### Hematoxylin–eosin (HE) staining

Histopathological examination of myocardial tissues was performed by HE staining (Beyotime). The left ventricle of the heart specimen was placed in 10% formaldehyde solution, dehydrated in an ethanol gradient, paraffin-embedded, cut into 4-μm sections, and stained with HE. The sections were then mounted and observed under a light microscope (Eclipse TE2000-U, Nikon Instruments Inc., Melville, NY, USA). The tissues were scored by myocardial histopathological assessment as follows: 0, no injury; 1, focal myocyte injury; 2, small multifocal degeneration with mild inflammatory cell infiltration; 3, extensive myofiber degeneration and/or diffuse inflammation; 4, necrosis with diffuse inflammation [15].

### Enzyme-linked immunosorbent assays (ELISA)

After the HF rats were generated, peripheral venous blood was drawn, and the serum was collected after centrifugation at 4℃ at 3,000 r/min for 10 min. ELISA kits (R&D Systems, Minneapolis, MN, USA) were used to test the levels of pro-inflammatory factors TNF-α (RTA00) and IL-6 (R6000B). The contents of rat myocardial injury markers were measured by rat cTnT ELISA Kit (E-EL-R0151c, Elabscience Biotechnology Co., Ltd., Wuhan, Hubei, China) and rat CK-MB ELISA Kit (E4608, BioVision, Inc., Exton, PA, USA).

### TUNEL assay

Myocardial tissues were fixed overnight in 10% formalin at 4 °C, sectioned (5 μm), and stained with a TUNEL kit (Roche Diagnostic Systems, Inc., branch, NJ, USA) as per the manufacturer's protocol to assess cardiomyocyte apoptosis in myocardial sections. The myocardial sections were incubated with 50 μL TUNEL mixture at 37 °C for 60 min and sealed with neutral resin. The generated sections were examined under a light microscope (Eclipse TE2000-U, Nikon), and the apoptotic rate was calculated as TUNEL-positive cells per field.

### Immunohistochemistry

Myocardial tissues were routinely paraffin-embedded and sectioned at 4 μm. The sections were first dewaxed and rehydrated. After blockage of endogenous peroxidase with 3% hydrogen peroxide solution, the sections were blocked with 100 μL of 5% BSA at 37 °C for 0.5 h. The sections were incubated overnight in 50 μL antibody against α-SMA (1:500, GTX100034, GeneTex) at 4 °C and with biotin-labeled secondary antibody goat anti-rabbit working solution (1:2000, ab205718, Abcam) at 37 °C for 0.5 h. Then, the sections were incubated with streptavidin-peroxidase solution (Solarbio, Beijing, China) for 0.5 h at 37 °C. Finally, the sections were treated with diaminobenzidine, counter-stained with hematoxylin for 30 s, dehydrated, fixed, observed and counted under a microscopy (Olympus Optical Co., Ltd., Tokyo, Japan).

### Chromatin immunoprecipitation (ChIP) assay

Cells in logarithmic growth phase were mixed with 1% formaldehyde for 10 min at room temperature to form deoxyribonucleic acid-protein crosslinks. The formed cross-links were fragmented by an ultrasonic device to break chromatin fragments into 200 to 1000 bp. The supernatant was collected by centrifugation at 13,000 rpm for 5 min at 4 °C and incubated overnight at 4 °C with rabbit antibodies to G9a (1:100, GTX129153, GeneTex) or H3K9me2 (1:100, GTX54102, GeneTex) with IgG (1:2000, ab172730, Abcam) as a control antibody. Endogenous DNA–protein complexes were precipitated by protein agarose/agarose gels. The supernatant was discarded following centrifugation. The crosslinking was reversed overnight at 65 °C. Then, the DNA fragments were purified by phenol/chloroform extraction. Finally, BDNF promoter expression in DNA generated by de-crosslinking was analyzed by qPCR.

### Statistics

The experiments were performed at least three times. Statistical analyses were conducted using SPSS 22.0 (IBM, Chicago, IL, USA). Values are mean ± SD. Two-group comparisons were performed by unpaired *t* test. One-way and two-way ANOVA with post hoc Tukey testing was applied for multiple comparison purposes. For all analyses, a minimum value of *p* < 0.05 was considered significant; when present, a *p* value < 0.01 or 0.001 was specified.

## Results

### G9a is upregulated in both HF patients and OGD-treated cell models

**Fig. 1:**
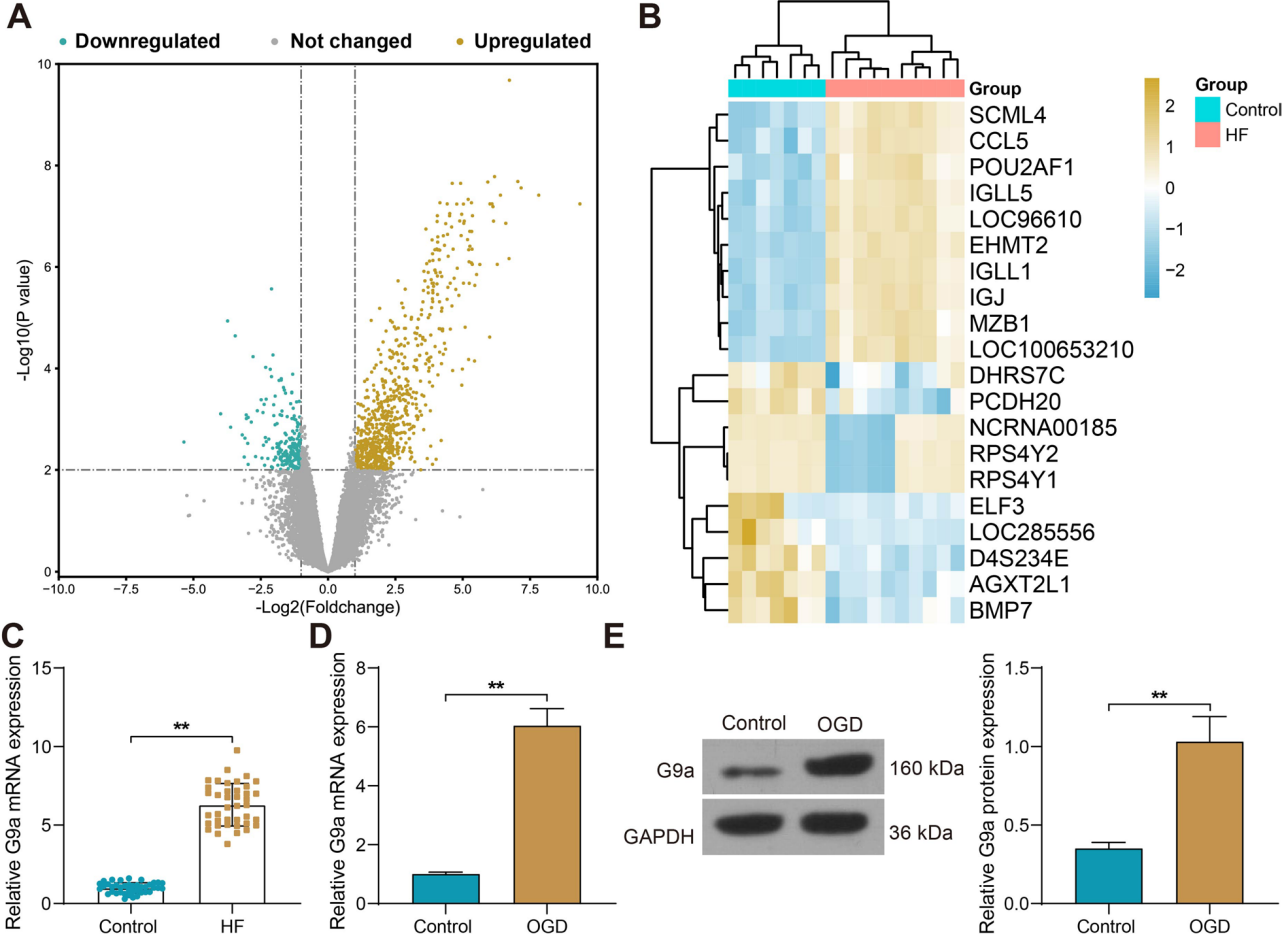
G9a is upregulated in both HF patients and OGD-treated cell models. **A** A total of 487 differentially expressed genes by screening in the GSE120895 dataset. **B** Heatmap analysis of the top 20 significantly differentially expressed mRNAs. **C** RT-qPCR analysis of G9a mRNA expression in collected HF patient sera (n = 40) and healthy subject sera (n = 40). **D**, **E** Expression of G9a in OGD-treated cell models and controls detected by RT-qPCR (**D**) and Western blot (**E**) (Uncropped protein blots are provided in the Additional file 1). Data are expressed as mean ± SD. Each experiment was repeated at least three times. A comparison of data from 2 groups was performed by an unpaired *t* test. **p < 0.01

We obtained mRNA data on HF patients in the GEO database GSE120895, including human endomyocardial biopsies of 10 patients with dilated cardiomyopathy and 7 individuals with normal LVEF. By setting |Fold Change|> 2 and Adj *p* value values < 0.01 as screening thresholds, 39 genes were significantly downregulated and 448 genes were significantly overexpressed in HF (Fig. 1A). The top 10 significantly downregulated and upregulated genes in the database were selected to plot the heatmap. To explore epigenetic phenomena in HF, we selected EHMT2 (also known as G9a) as the subject of study (Fig. 1B). Our RT-qPCR analysis of sera collected from HF patients and healthy subjects revealed that G9a was overexpressed in the sera of HF patients (Fig. 1C). We then constructed a HF cell model by treating H9C2 cells with OGD. Both mRNA and protein expression profiles of G9a were significantly elevated in the OGD cells compared to control cells (Fig. 1D, E).

### Knockdown of G9a inhibits apoptosis and injury in OGD-treated H9C2 cells

**Fig. 2:**
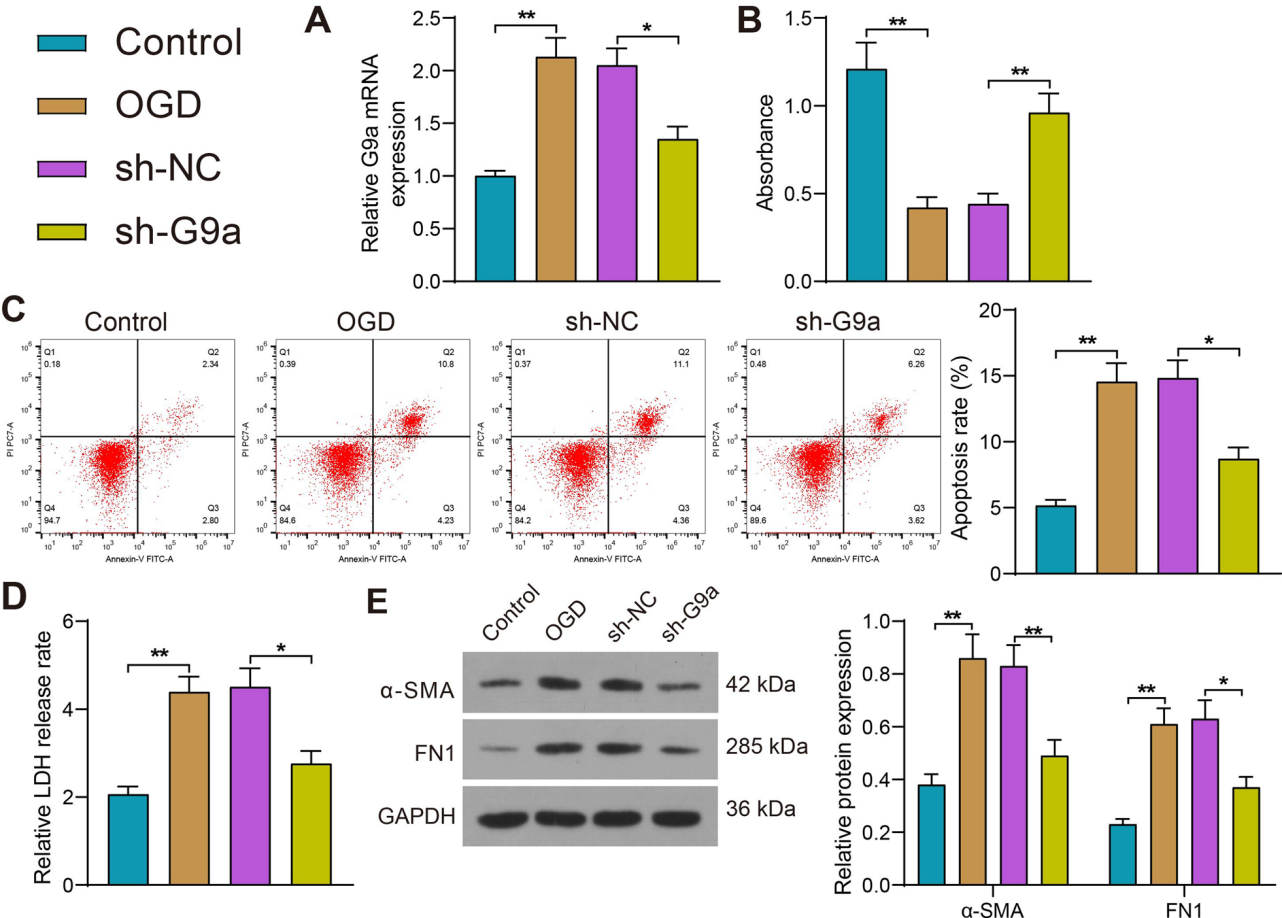
Knockdown of G9a inhibits apoptosis and injury in OGD-treated cardiomyocytes. **A** The mRNA expression of G9a in cells detected by RT-qPCR. **B** The proliferative activity of cells measured by CCK8 assay. **C** The apoptotic activity of cells measured using flow cytometry. **D** Detection of cytotoxicity of cells by LDH cytotoxicity kits. **E** The levels of fibrosis-related factors α-SMA and FN1 in cells examined using Western blot (Uncropped protein blots are provided in the Additional file 1). Data are expressed as mean ± SD. Each experiment was repeated at least three times. Three or more groups were compared by one-way or two-way ANOVA and Tukey's post hoc test. *p < 0.05, **p < 0.01

G9a was knocked down to investigate its effect on OGD-treated H9C2 cells. The RT-qPCR results confirmed that the expression of G9a was significantly downregulated in H9C2 cells after transfection with sh-G9a (Fig. 2A). Studies of the proliferative activity of the cells showed that OGD significantly reduced H9C2 cell viability, while knockdown of G9a significantly increased H9C2 cell viability (Fig. 2B). Flow cytometry assay showed that OGD treatment significantly increased the proportion of apoptotic H9C2 cells, but sh-G9a treatment significantly downregulated the number of apoptotic H9C2 cells (Fig. 2C). OGD-induced cytotoxicity in H9C2 cells was detected using the LDH cytotoxicity kit, and the results obtained were consistent with flow cytometry (Fig. 2D). In addition, we examined the levels of cellular fibrosis-associated factors α-SMA and FN1 in H9C2 cells by Western blot, which showed that OGD treatment significantly increased the levels of α-SMA and FN1, but sh-G9a treatment significantly inhibited the fibrosis of H9C2 cells (Fig. 2E). Thus, our findings suggest that knockdown of G9a attenuates OGD-induced H9C2 cell injury by increasing cell viability and reducing apoptosis and cytotoxicity.

### Inhibition of G9a expression attenuates myocardial injury in rats with HF

**Fig. 3:**
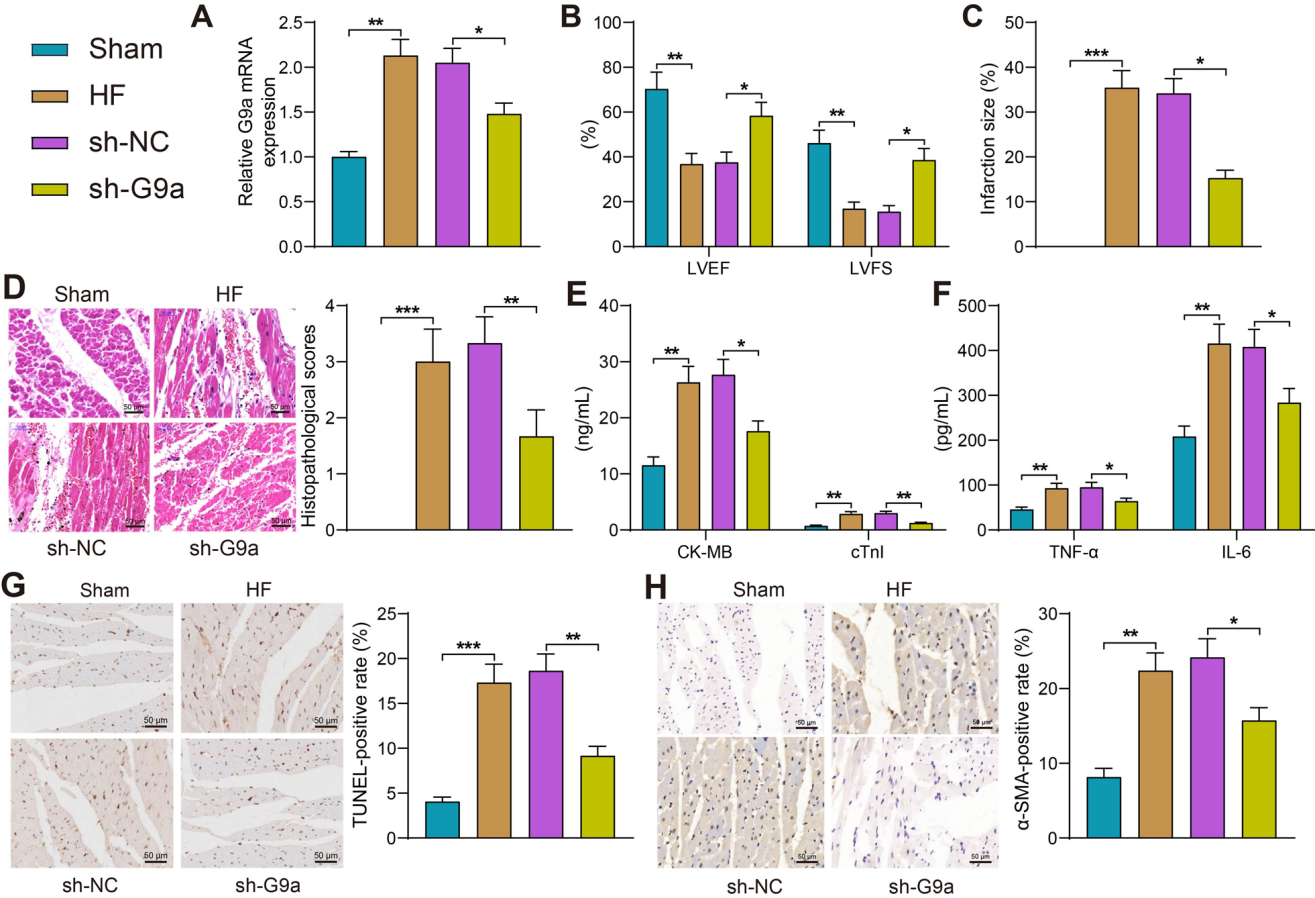
Inhibition of G9a attenuates the myocardial injury in rats with HF. **A** Detection of G9a expression in rats by RT-qPCR. **B** Echocardiographic assessment of cardiac function in rats. **C** Myocardial damage in rats examined using TTC staining. **D** The pathological changes in the myocardial tissues of rats measured using HE staining. **E** Detection of CK-MB and cTnI in the serum of rats measured using ELISA. **F** Changes of inflammatory factors IL-6 and TNF-α in rats detected by ELISA. **G** Apoptosis in myocardial tissues of rats measured using TUNEL assay. **H** Changes of α-SMA in myocardial tissues of rats by immunohistochemistry. Data are expressed as mean ± SD (n = 6). Three or more groups were compared by one-way or two-way ANOVA and Tukey's post hoc test. *p < 0.05, **p < 0.01, ***p < 0.001

To investigate the effect of G9a on HF rats, we silenced G9a by injection of lentivirus carrying sh-G9a into HF rats. We found that G9a expression was significantly higher in myocardial tissues of HF rats and decreased significantly after injection of lentivirus carrying sh-G9a (Fig. 3A). We then tested the cardiac function of rats and found that LVEF and LVFS were significantly reduced in HF rats, which were increased after depletion of G9a (Fig. 3B). TTC staining showed that the infarct area was significantly larger in HF rats compared with the sham-operated rats, and sh-G9a treatment significantly reduced the infarct area (Fig. 3C). It was displayed by HE staining that the cardiomyocytes in HF rats were disorganized with significantly enlarged intercellular gaps and elevated myocardial histopathological scores, and downregulation of G9a alleviated cardiomyocyte injury in rats (Fig. 3D). The levels of cardiac injury markers CK-MB and cTnI in rat serum was evaluated by ELISA. The levels of CK-MB and cTnI were significantly enhanced in the serum of HF rats, while sh-G9a treatment decreased the expression of injury markers (Fig. 3E). Consistently, sh-G9a treatment lowered the levels of pro-inflammatory factors induced by LAD surgery (Fig. 3F). Apoptosis was significantly promoted in cardiomyocytes of HF rats using TUNEL assay, and knockdown of G9a reduced apoptosis in cardiomyocytes (Fig. 3G). Finally, immunohistochemical staining showed a significant surge in the intensity of the fibrosis-associated factor α-SMA staining in the myocardial tissues of HF rats and a decline in the expression of α-SMA after silencing of G9a (Fig. 3H). Based on these results, we conclude that inhibition of G9a expression significantly suppresses inflammation, reduces the proportion of apoptotic cells and the level of fibrosis in myocardial tissues, and attenuates myocardial injury in HF rats.

### Silencing of G9a promotes BDNF expression by repressing H3K9me2 modification of the BDNF promoter

**Fig. 4:**
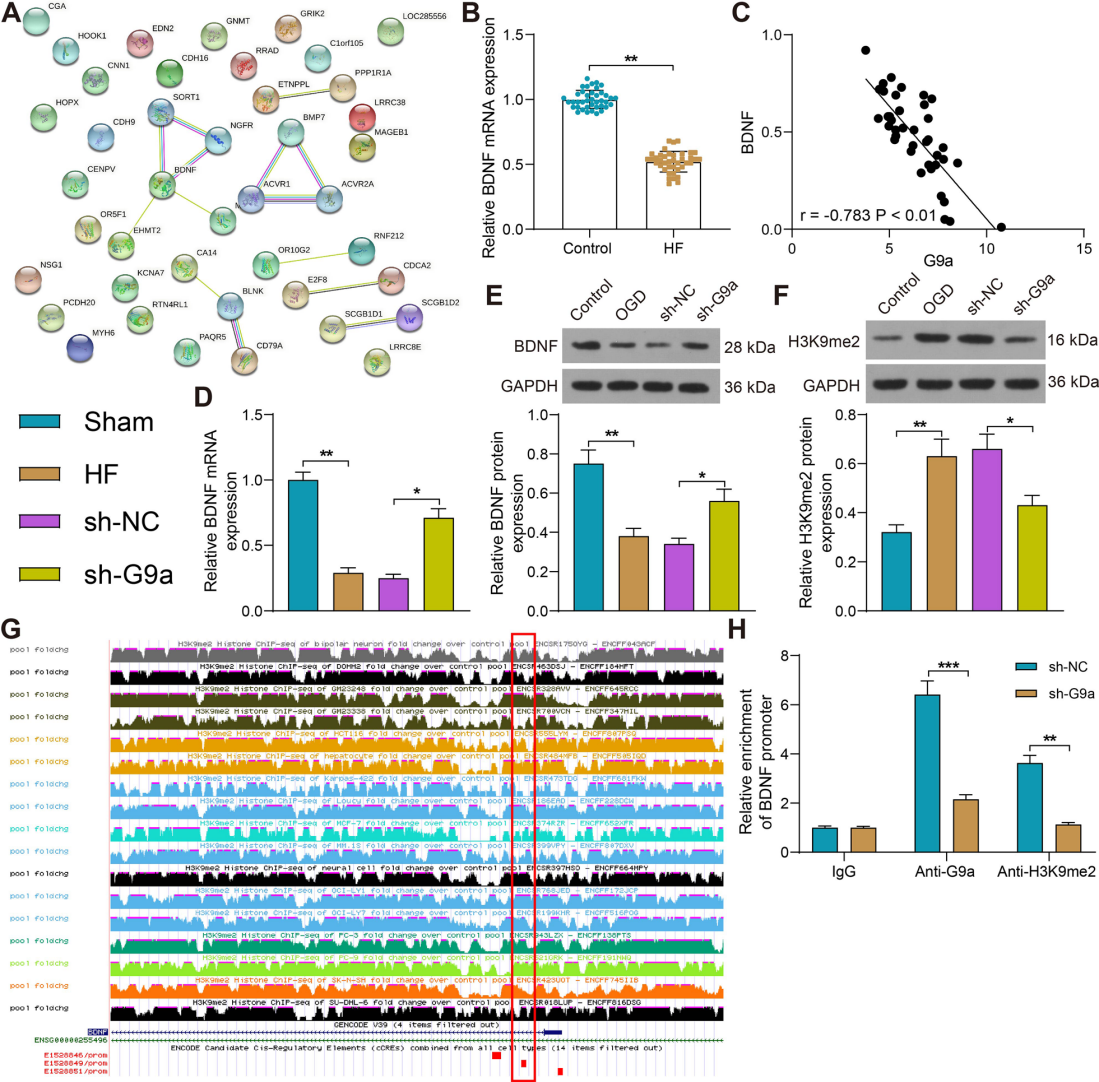
Silencing of G9a promotes BDNF expression by repressing H3K9me2 modification of the BDNF promoter. **A** Interaction of G9a with downregulated genes visualized using PPI network. **B** Detection of BDNF expression in sera of HF patients (n = 40) and healthy subjects (n = 40) by RT-qPCR. **C** Pearson’s analysis of the correlation between the expression of G9a and BDNF. **D** Detection of BDNF expression in myocardial tissues of rats (n = 6) by RT-qPCR. **E** Analysis of BDNF expression in cells by Western blot (Uncropped protein blots are provided in the Additional file 1). **F** The expression of H3K9me2 in cells by Western blot (Uncropped protein blots are provided in the Additional file 1). **G** ChIP-seq data of H3K9me2 enrichment peaks on BDNF promoter (n = 17). **H** Detection of BDNF promoter enriched by Anti-G9a or Anti-H3K9me2 by ChIP-qPCR. Data are expressed as mean ± SD. Each experiment was repeated at least three times unless otherwise indicated. A comparison of data from 2 groups was performed by an unpaired *t* test, while 3 or more groups were compared by one-way or two-way ANOVA and Tukey's post hoc test. *p < 0.05, **p < 0.01, ***p < 0.001

We constructed protein–protein interactions (PPI) networks to retrieve genes repressed under the action of G9a and found a correlation between BDNF and G9a. In addition, BDNF as a gene downregulated in the myocardial tissues of HF patients has interaction with several proteins and may be a key gene in the process of HF (Fig. 4A). BDNF expression was detected by RT-qPCR on sera collected from HF patients and healthy subjects and was found to be significantly reduced in our cohort (Fig. 4B). Correlation analysis of G9a and BDNF expression revealed a significant negative correlation between the two genes (Fig. 4C). Moreover, BDNF mRNA expression was significantly lower in the myocardial tissues of HF rats, and significantly higher after knockdown of G9a (Fig. 4D). Consistent results were observed using western blot in OGD-treated H9C2 cells as well (Fig. 4E). Because G9a can repress gene expression through H3K9me2 [16], we used Western blot assay to examine the expression of H3K9me2 in H9C2 cells. It was found that its expression was significantly elevated in OGD-treated H9C2 cells, which was significantly reduced in cells treated with sh-G9a (Fig. 4F). ChIP-seq predicted an H3K9me2 binding peak on the BDNF promoter (Fig. 4G). The results of ChIP-qPCR assay showed that the enrichment ability of G9a and H3K9me2 on the BDNF promoter was significantly reduced after silencing of G9a (Fig. 4H). The above results suggest that silencing of G9a inhibits the H3K9me2 modification of BDNF promoter and promotes the expression of BDNF.

### Silencing of BDNF partially reverses the effect of sh-G9a on mitigating apoptosis and injury in cardiomyocytes by inhibiting the TrkB signaling

**Fig. 5:**
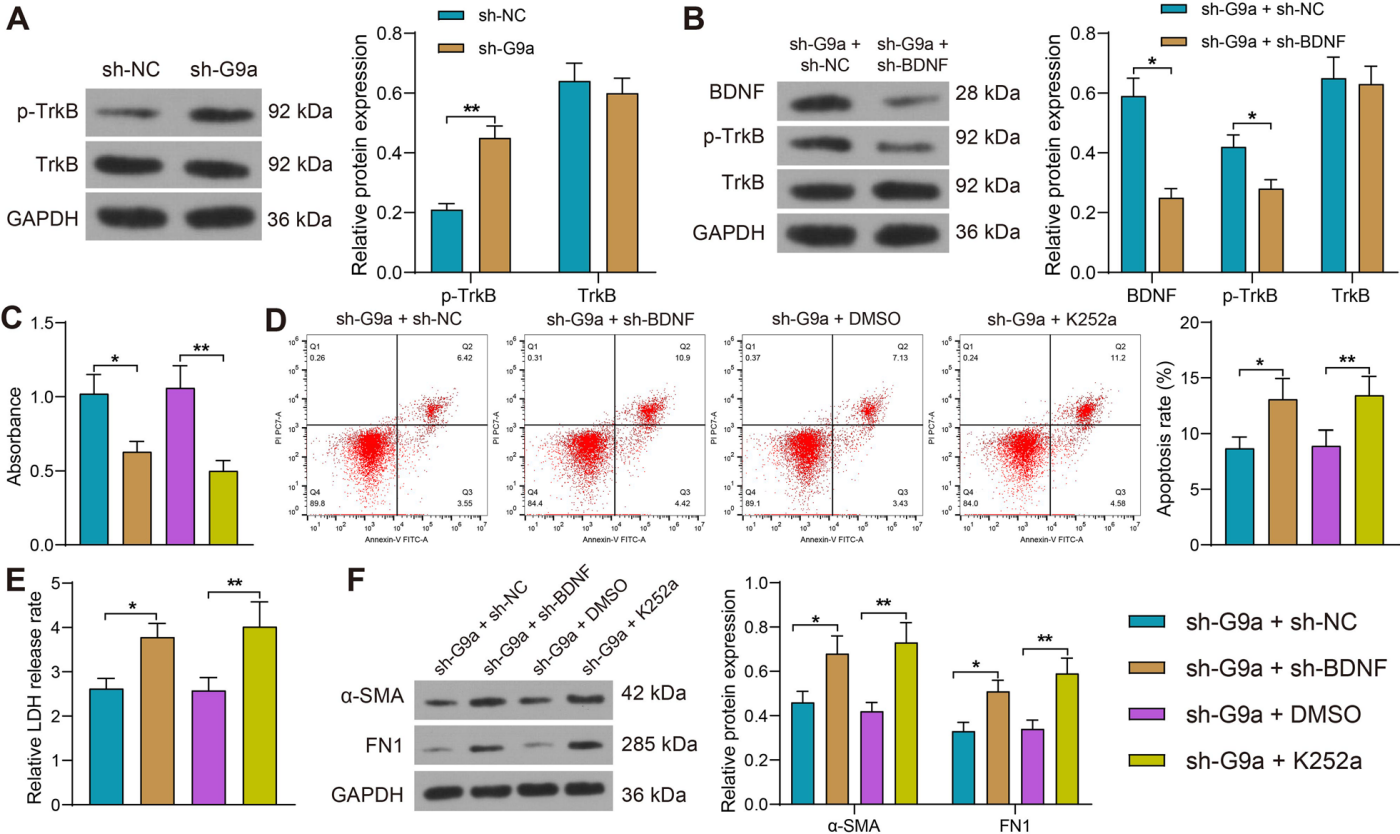
Silencing of BDNF partially reverses the effect of sh-G9a on alleviating cardiomyocyte apoptosis and injury by inhibiting the TrkB signaling pathway. **A** Detection of extent of TrkB phosphorylation in H9C2 cells transfected with sh-G9a by Western blot (Uncropped protein blots are provided in the Additional file 1). **B** The protein levels of BDNF, TrkB and p-TrkB in cells transfected with sh-G9a + sh-BDNF expression by Western blot (Uncropped protein blots are provided in the Additional file 1). **C** The proliferative activity of cells measured by CCK8 assay. **D** The apoptotic activity of cells measured using flow cytometry. **E** Detection of cytotoxicity of cells by LDH cytotoxicity kits. **F** The levels of fibrosis-related factors α-SMA and FN1 in cells examined using Western blot (Uncropped protein blots are provided in the Additional file 1). Data are expressed as mean ± SD. Each experiment was repeated at least three times. Three or more groups were compared by one-way or two-way ANOVA and Tukey's post hoc test. *p < 0.05, **p < 0.01

The extent of TrkB phosphorylation was found to be significantly increased in the sh-G9a-transfected cells by Western blot assay (Fig. 5A). We further inhibited BDNF expression in H9C2 cells by transfection of sh-BDNF in the presence of sh-G9a, and Western blot analysis exhibited that the protein levels of both BDNF and p-TrkB were reduced (Fig. 5B). Meanwhile, we blocked the TrkB activation using K252a in H9C2 cells in the presence of sh-G9a with DMSO as control. Proliferative activity of cardiomyocytes was detected by CCK8, and both downregulation of BDNF and K252a inhibited cellular activity (Fig. 5C). Flow cytometry results showed that downregulation of BDNF and K252a reversed the effect of sh-G9a and elevated the proportion of apoptotic cells (Fig. 5D). Inhibition of BDNF expression and BDNF/TrkB binding contributed to higher cytotoxicity by LDH cytotoxicity kit assay (Fig. 5E). In addition, we found increased protein levels of α-SMA and FN1 in cells after knocking down BDNF or blocking BDNF/TrkB binding by Western blot analysis (Fig. 5F).

### Blockade of BDNF/TrkB reverses sh-G9a action to exacerbate myocardial injury in HF rats

**Fig. 6:**
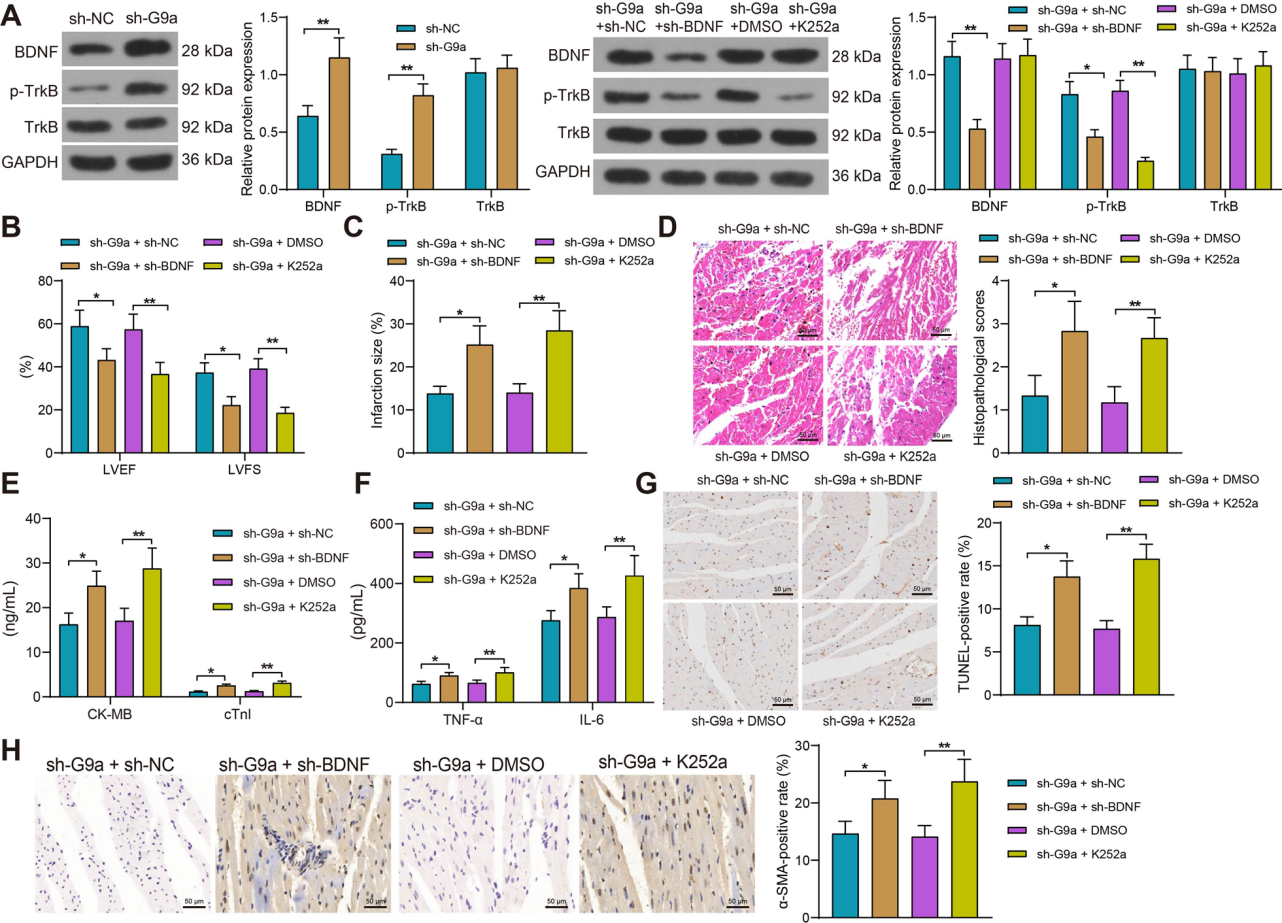
Silencing of BDNF partially reverses the effect of sh-G9a on alleviating myocardial injury in rats with HF by inhibiting the TrkB signaling pathway. **A** The protein expression BDNF and the extent of TrkB phosphorylation in rat myocardial tissues by Western blot (Uncropped protein blots are provided in the Additional file 1). **B** Echocardiographic assessment of cardiac function in rats. **C** Myocardial damage in rats examined using TTC staining. **D** The pathological changes in the myocardial tissues of rats measured using HE staining. **E** Detection of CK-MB and cTnI in the serum of rats measured using ELISA. **F** Changes of inflammatory factors IL-6 and TNF-α in rats detected by ELISA. **G** Apoptosis in myocardial tissues of rats measured using TUNEL assay. **H** Changes of α-SMA in myocardial tissues of rats by immunohistochemistry. Data are expressed as mean ± SD (n = 6). Three or more groups were compared by one-way or two-way ANOVA and Tukey's post hoc test. *p < 0.05, **p < 0.01

sh-BDNF or K252a was also injected into sh-G9a-injected rats to impair BDNF expression or BDNF/TrkB binding, respectively. The results of western blot in rats showed that depletion of G9a restored BDNF protein expression and TrkB phosphorylation in myocardial tissues, whereas sh-BDNF reversed the effect of sh-G9a to reduce BDNF protein expression and TrkB phosphorylation. K252a only inhibited TrkB phosphorylation, with no significant influence in BDNF expression (Fig. 6A). LVEF and LVFS were significantly reduced in HF rats after BDNF reduction and K252a application, and cardiac function was attenuated in rats (Fig. 6B). TTC staining revealed that BDNF downregulation and inhibition of BDNF/TRKB binding diminished the effect of sh-G9a and increased myocardial infarct area in rats (Fig. 6C). At the same time, HE staining showed downregulation of BDNF and K252a aggravated damage to myocardial tissues in rats (Fig. 6D). Levels of cardiac injury markers CK-MB and cTnI and inflammatory factors IL-6 and TNF-α were also significantly elevated after BDNF downregulation and K252a application (Fig. 6E, F). Detection of cardiomyocyte apoptosis showed that sh-BDNF and K252a treatment significantly exacerbated cardiomyocyte apoptosis in HF rats (Fig. 6G). Immunohistochemical staining indicated accelerated myocardial fibrosis in response to sh-BDNF and K252a in the presence of sh-G9a (Fig. 6H). As shown above, both the downregulation of BDNF expression and the inhibition of BDNF/TrkB binding reversed the effects of sh-G9a and significantly exacerbated myocardial injury in rats.

## Discussion

HF, a syndrome caused by a complex genetic predisposition and various environmental factors, is a main cause of morbidity and mortality [17]. Epigenetics is essential for many essential processes in biology, but its significance to the development of HF remains unclear [18, 19]. Histone modifiers, such as histone methyltransferases and histone deacetylases, catalyze the modification of histone proteins to change the histone-DNA contacts, thereby controlling the availability of genomic loci to transcriptional mediation or other regulation [20]. It was found that epigenetics governs the transcription of pro-fibrotic genes in myocardial fibrosis by histone modification [21]. In this study, we demonstrate that depletion of G9a inhibited the H3K9me2 modification on the BDNF promoter and promoted the expression of BDNF. Moreover, BDNF restoration alleviated HF in rats via the phosphorylation of TrkB.

G9a was described to be participated in embryonic development, hypoxia, and DNA repair [16]. Chopra et al. revealed that the methyltransferase activity of G9a is hypoxia-inducible and thus present a novel avenue of low-oxygen signaling [22]. In the present study, we found that G9a was induced in OGD-exposed cells in addition to upregulation of G9a in the serum and myocardial tissues of patients with HF. Barcena-Varela et al. revealed that G9a expression was induced during mouse hepatic stellate cell activation, and G9a knockdown inhibited TGF-β1 fibrogenic responses in human hepatic stellate cells [23]. More relevantly, the combination G9a inhibitor and erythropoietin was superior to either one alone for protecting myocardium from acute myocardial infarction damage by suppressing the expression of inflammatory, anti-oxidative-stress, anti-ischemic biomarkers [24]. Here, we reported that knockdown of G9a not only alleviated cell injury by repressing cell cytotoxicity and fibrosis, but also ameliorated HF in rats by aborting inflammation and fibrosis.

Gupta-Agarwal et al. revealed that downregulation of G9a activity in the entorhinal cortex enhanced H3K9me2 in area CA1, leading to transcriptional silencing of the non-memory permissive gene COMT in the hippocampus [25]. We thus generated a PPI network to decipher the downstream key genes of G9a in HF. BDNF was revealed to be the most significant one. BDNF in serum of patients with coronary heart disease can be used as an effective biological indicator to monitor the degree of coronary heart disease and severity of coronary stenosis [26]. In addition, Takashio and Tschorn et al. found that plasma BDNF expression was reduced in patients with HF and associated with HF severity, suggesting that it might be a clinically valuable biomarker of HF [27, 28]. After G9a overexpression in vitro and in vivo, the enrichment levels of H3K9me2 in the promoter region of BDNF were augmented, whereas the levels of BDNF were decreased, along with damaged neurons and impaired learning and memory abilities of rats with hypoxic-ischemic encephalopathy [29]. There findings were largely in line with our observation where enrichment of BDNF promoters by G9a and H3K9me2 was significantly reduced after silencing of G9a in H9C2 cells.

It has been reported by Becker et al. that BDNF/TrkB pathway is blocked in the dorsal medial nucleus tractus solitarius during chronic HF, which offers a new mechanism for understanding the central alterations that lead to baroreflex desensitization in chronic HF [30]. Here, the extent of TrkB phosphorylation was found to be activated in the cells treated with sh-G9a, which was partially reduced by further knockdown of BDNF. Matsumoto et al. showed that the beneficial effects of BDNF on the exercise capacity of HF mice were elicited by enhancing fatty acid oxidation [31]. More importantly, BDNF augmented cell viability, repressed apoptosis and DNA damage of H9C2 cells in vitro and in vivo [32, 33]. Here, the results of our rescue experiments exhibited that depletion of BDNF and the TrkB inhibitor led to enhanced cell cytotoxicity and fibrosis in the presence of sh-G9a. Activation of cardiac TrkB by its small molecular agonist 7,8-dihydroxyflavone could suppress doxorubicin-induced cardiotoxicity and myocardial ischemia [34, 35]. The data of our rescue experiments also showed that sh-BDNF and K252a accentuated myocardial injury and fibrosis in rats with HF in the presence of sh-G9a. Rats subjected to 8 weeks of treadmill running exhibited increased myocardial angiogenesis and improved cardiac function, which was deteriorated by K252a [14].

## Conclusion

In conclusion, silencing of G9a improves cardiac function, including LVEF and LVFS, reduces infarct size and alleviates fibrosis by restoring BDNF expression and the subsequent TrkB activation. These findings suggest that activation of the BDNF/TrkB axis by G9a silencing is a promising target to combat HF.

## Supplementary Information

**Additional file 1**. Full-length gels and blots.

## Data Availability

All the data generated or analyzed during this study are included in this published article.

## Abbreviations

HF: Heart failure
G9a: Euchromatic histone lysine methyltransferase
OGD: Oxygen–glucose deprivation
H3K9me2: Dimethylation of H3 lysine 9
BDNF: Brain-derived neurotrophic factor
TrkB: Tropomyosin-related kinase receptor B
DMEM: Dulbecco's modified Eagle's medium
FBS: Fetal bovine serum
sh: Short hairpin RNA
DMSO: Dimethylsulfoxide
LAD: Left anterior descending
RT-qPCR: Reverse transcription quantitative polymerase chain reaction
GAPDH: Glyceraldehyde-3-phosphate dehydrogenase
BSA: Bovine serum albumin
CCK-8: Cell counting kit
OD: Optical density
FITC: Fluorescein isothiocyanate
PI: Propidium iodide
LDH: Lactate dehydrogenase
TTC: Triphenyltetrazolium chloride
ANAR: Area not at risk
AAR: Area at risk
INF: Infarcted
HE: Hematoxylin–eosin
ELISA: Enzyme-linked immunosorbent assays
TUNEL: TdT-mediated dUTP nick end labeling
ChIP: Chromatin immunoprecipitation
