# Supplementary material for: H3K9me2 regulation of BDNF expression via G9a partakes in the progression of heart failure
Source: BMC Cardiovasc Disord. 2022 Apr 19;22:182. doi: 10.1186/s12872-022-02621-w (PMC9020036; doi:10.1186/s12872-022-02621-w)
Supplement: Supplementary file 1 — Additional file 1. Full-length gels and blots. [file 12872_2022_2621_MOESM1_ESM.docx]

**Additional file 1**: Full-length gels and blots.

**Figure 1E**

**
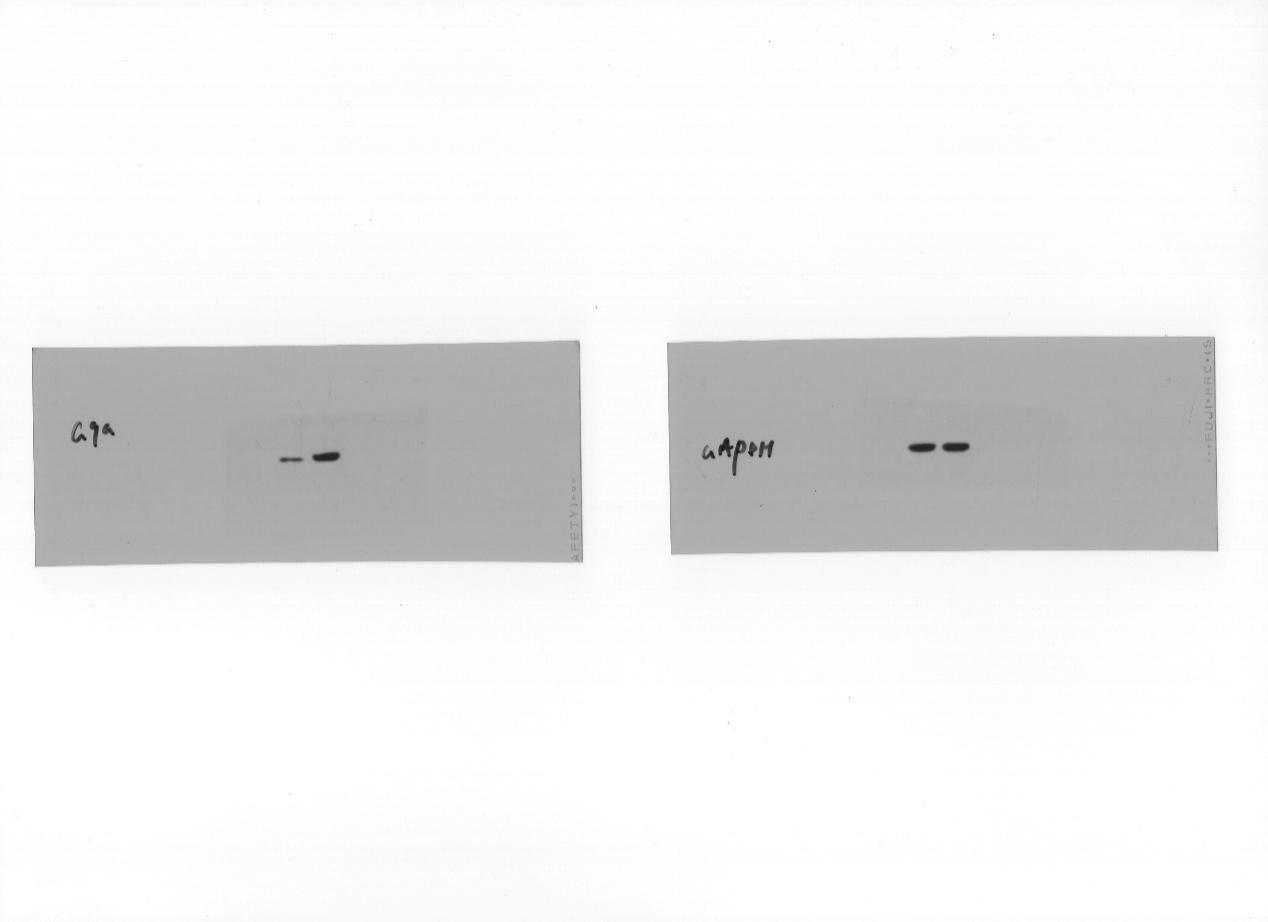
**

**Figure 2E**

**
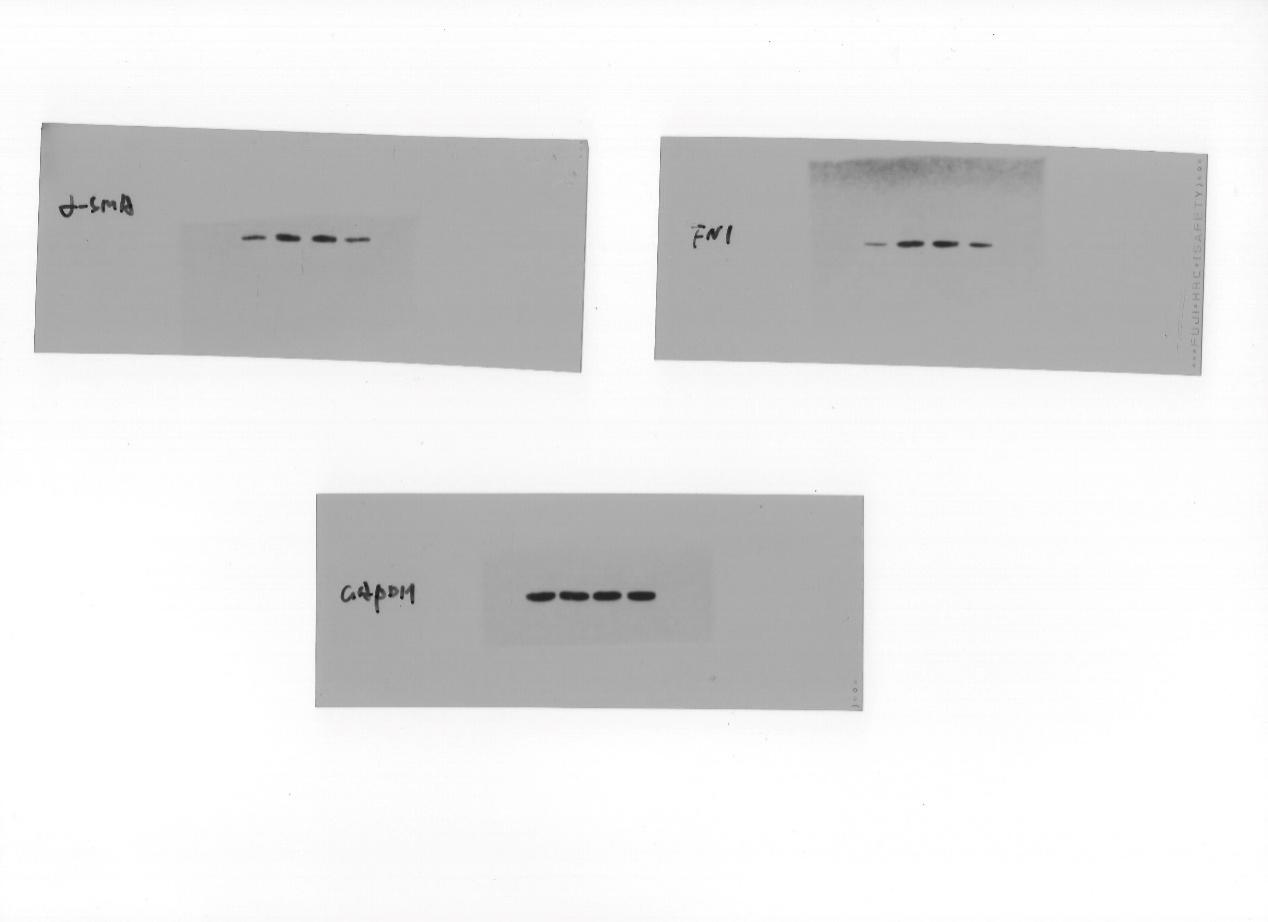
**

**Figure 4E**

**
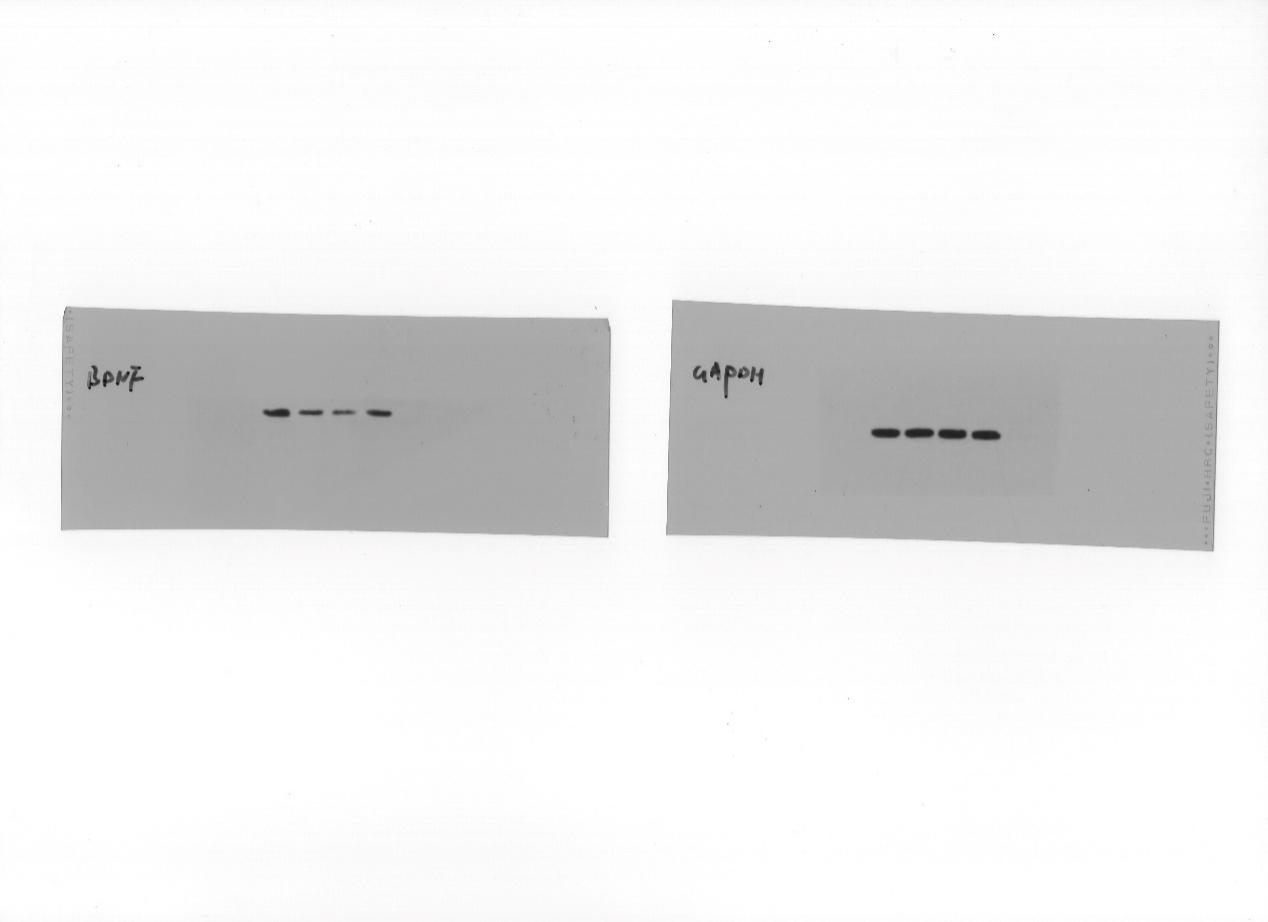
**

**Figure 4F**

**
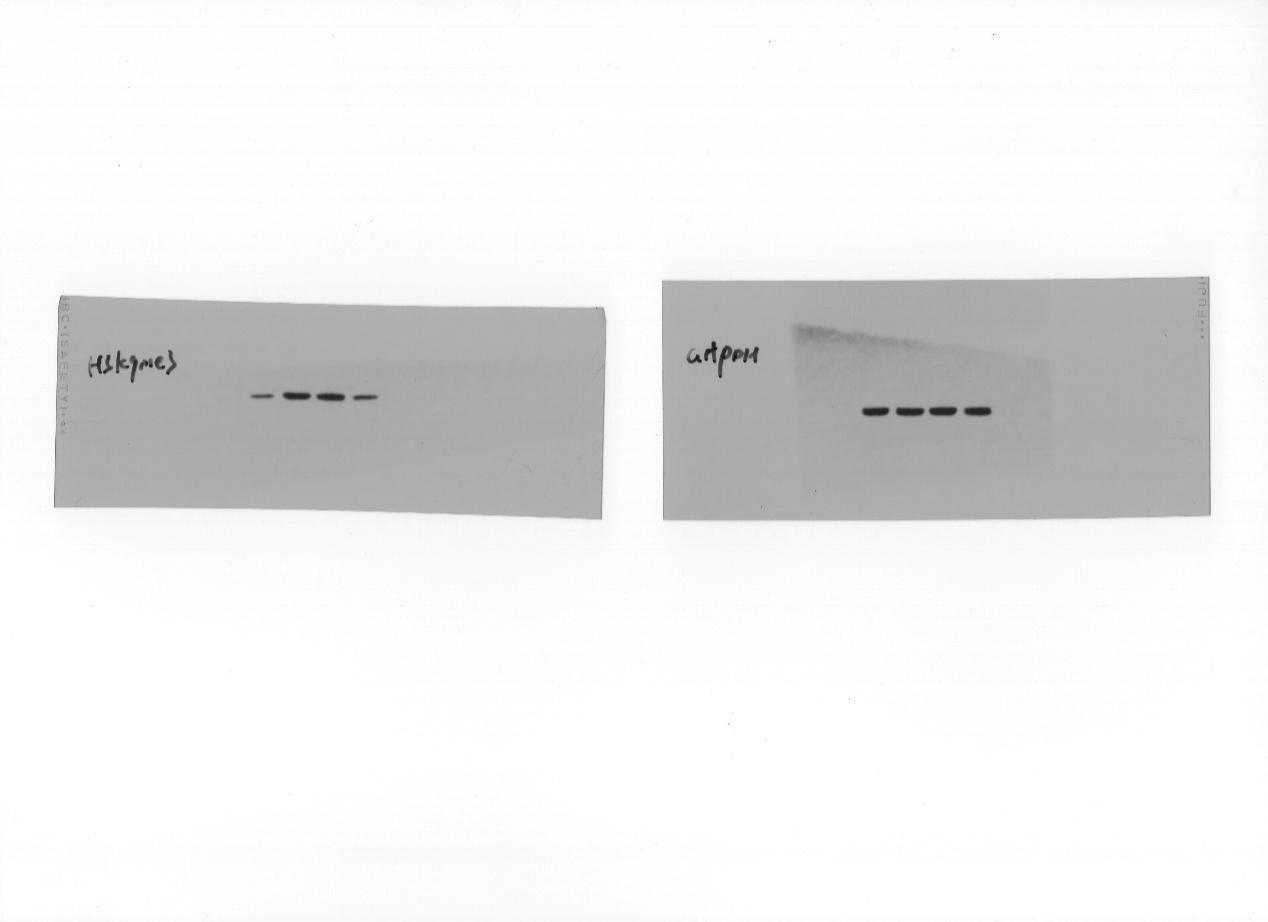
**

**Figure 5A**

**
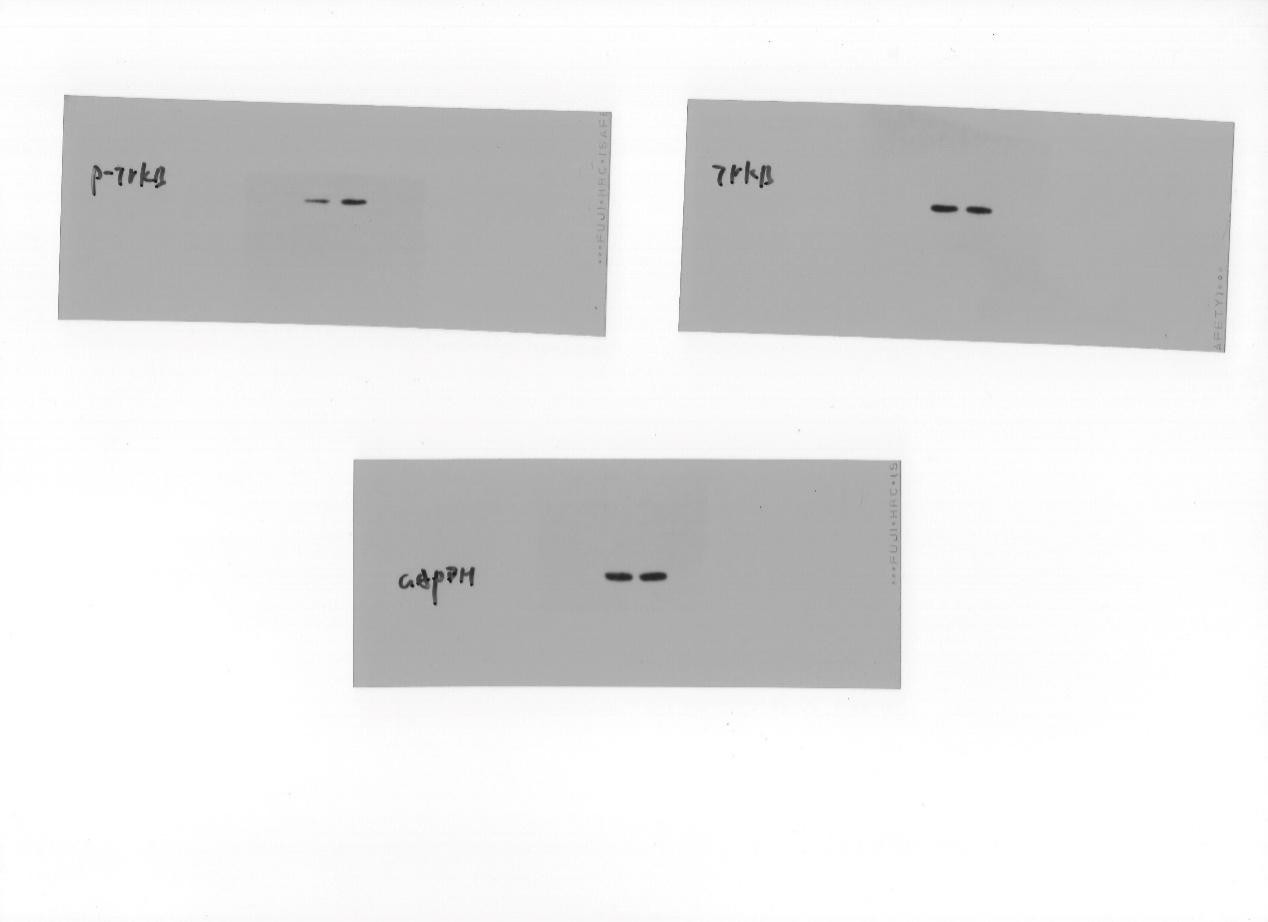
**

**Figure 5B**

**
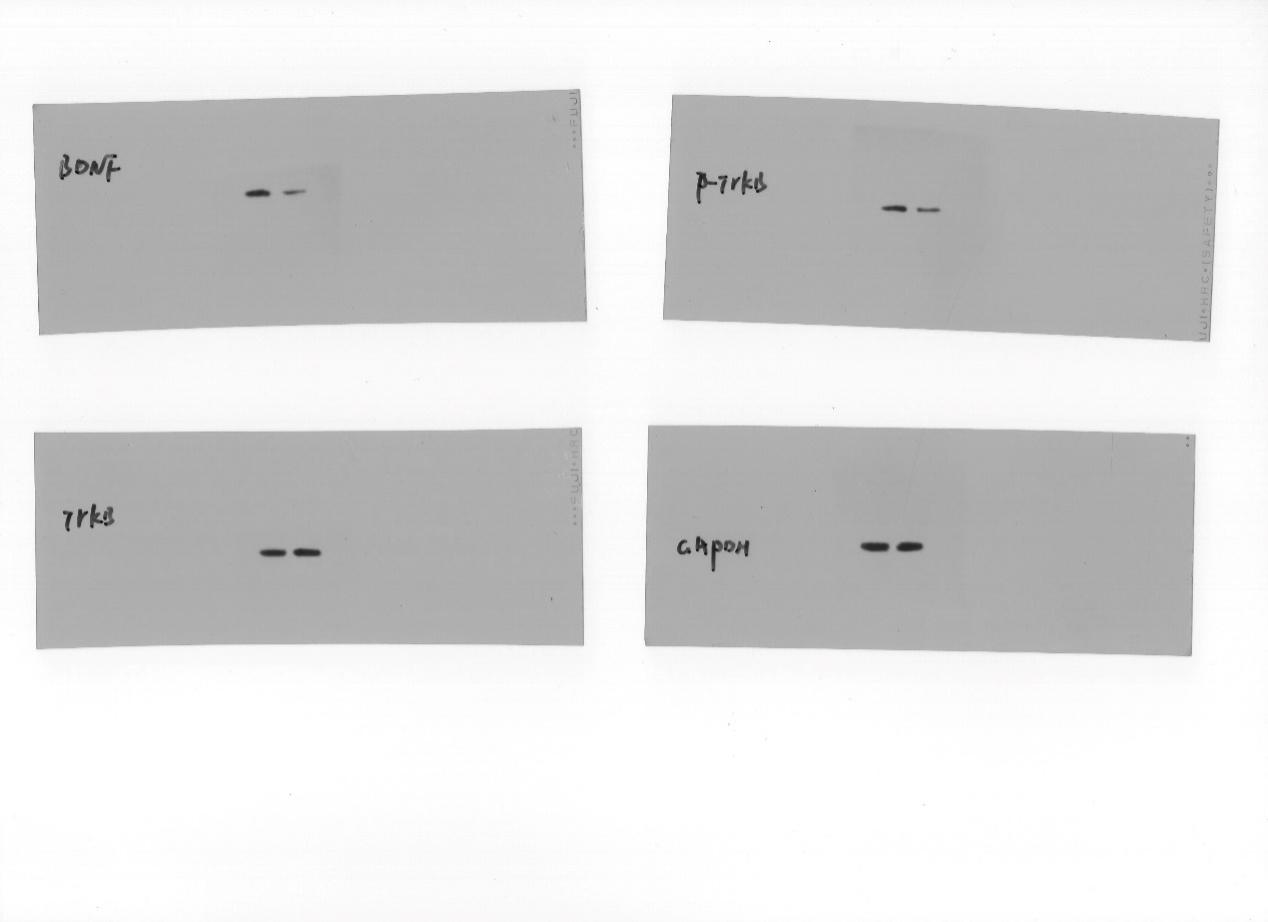
**

**Figure 5F**

**
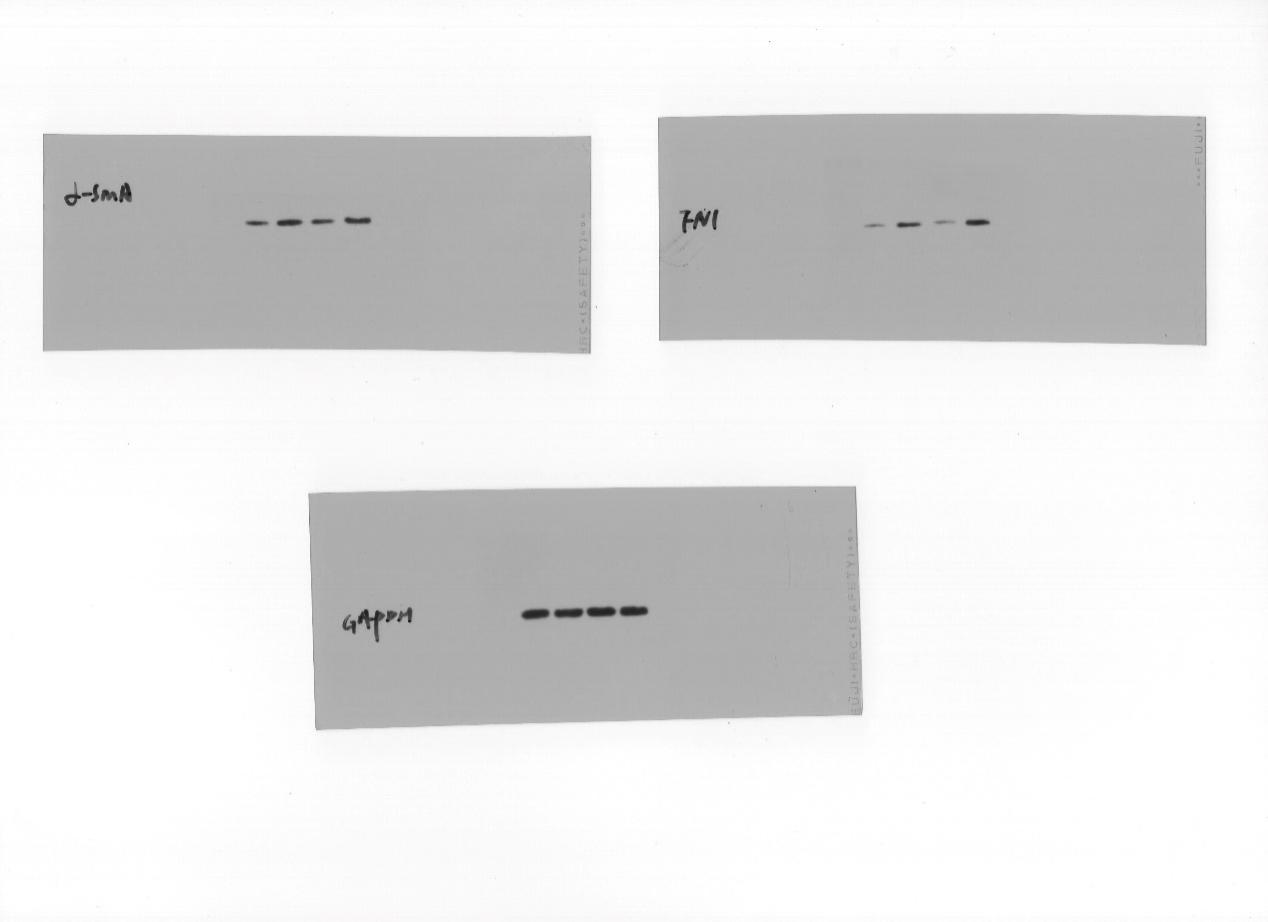
**

**Figure 6A-1**

**
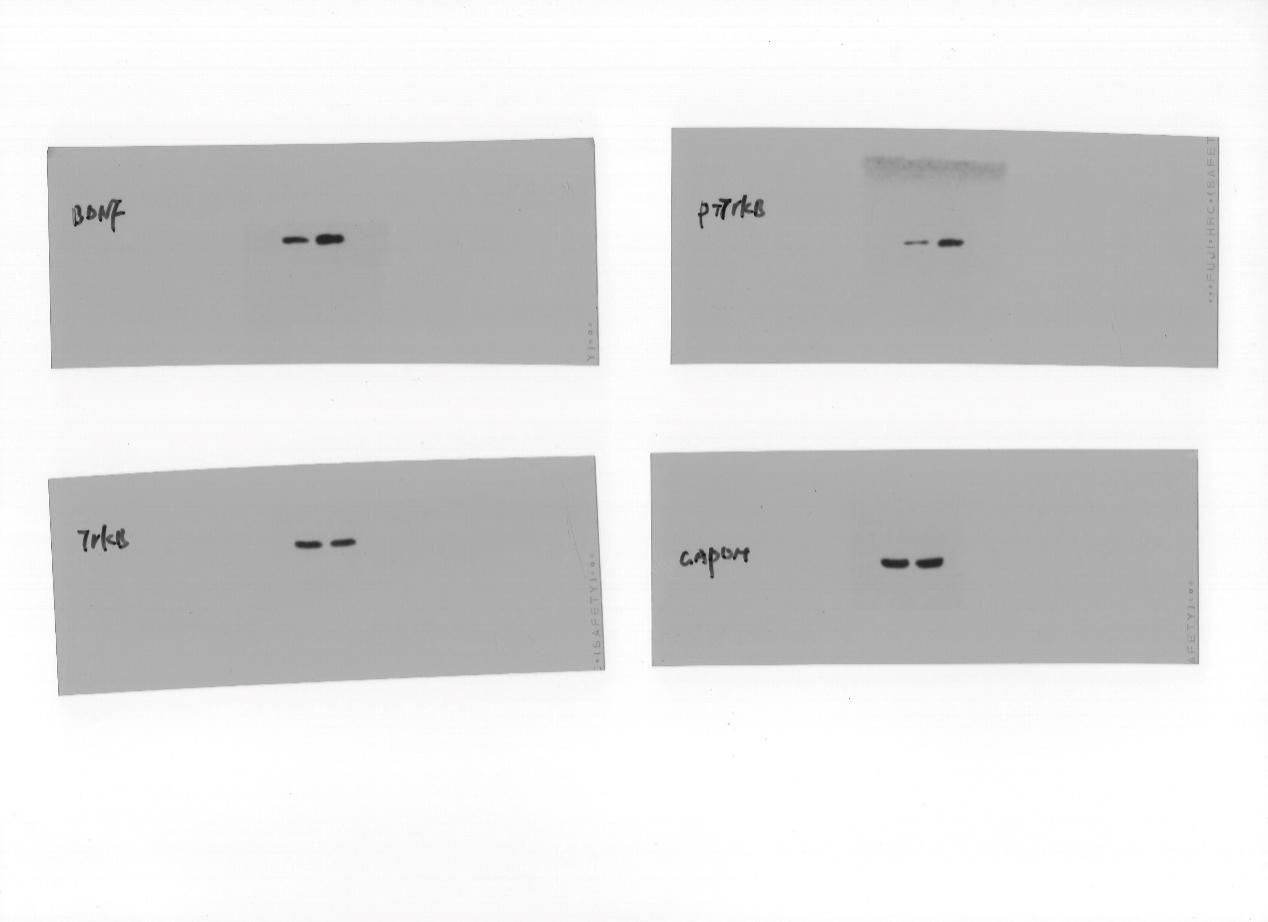
**

**Figure 6A-2**

**
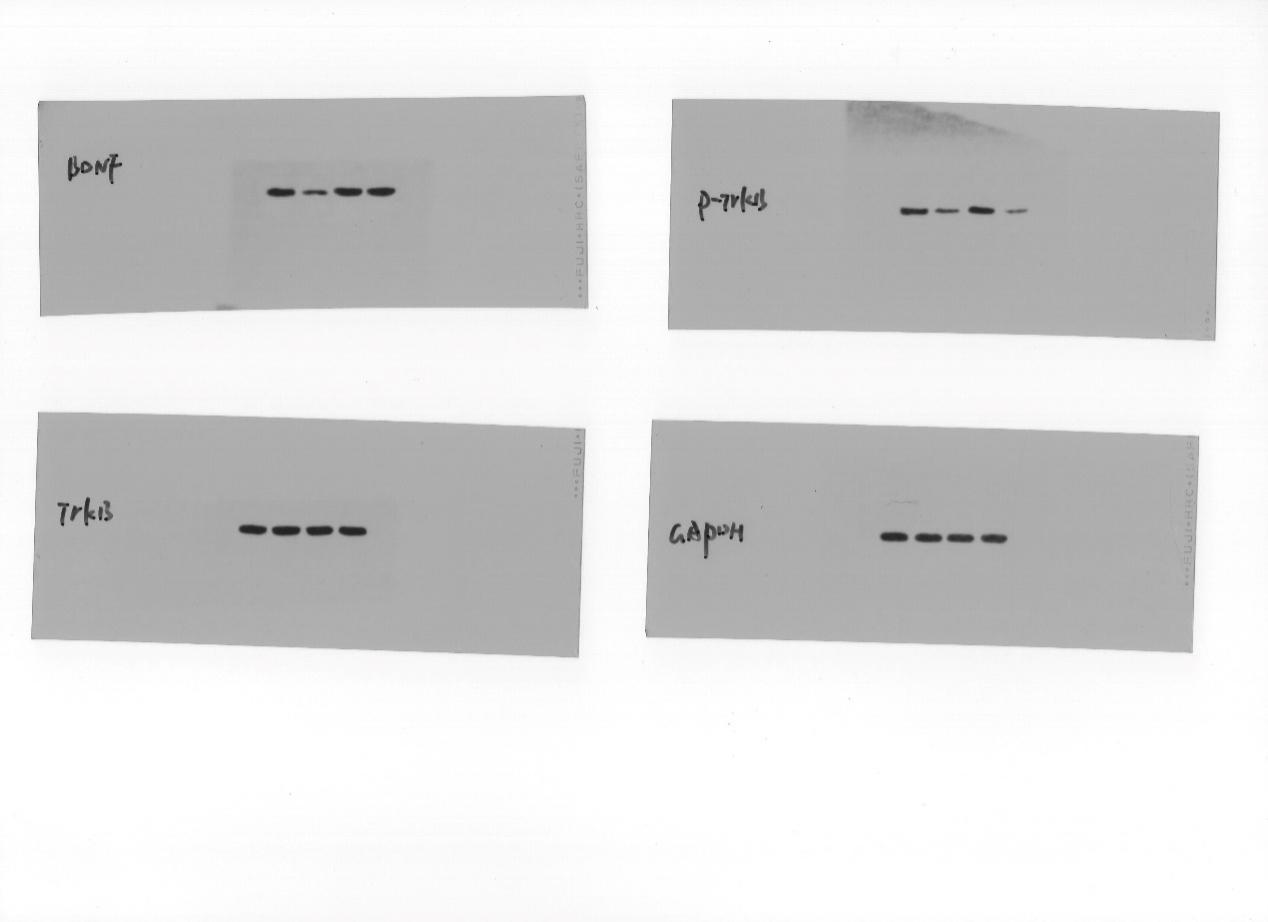
**
